# Resveratrol and Curcumin for Chagas Disease Treatment—A Systematic Review

**DOI:** 10.3390/ph15050609

**Published:** 2022-05-15

**Authors:** Carlos Henrique Lima Imperador, Cauê Benito Scarim, Priscila Longhin Bosquesi, Juliana Romano Lopes, Augusto Cardinalli Neto, Jeanine Giarolla, Elizabeth Igne Ferreira, Jean Leandro dos Santos, Chung Man Chin

**Affiliations:** 1 Advanced Research Center in Medicine (CEPAM), School of Medicine, Union of the Colleges of the Great Lakes (UNILAGO), Sao Jose do Rio Preto 15030-070, SP, Brazil; carloshimperador@gmail.com (C.H.L.I.); priscila.longhin@unilago.edu.br (P.L.B.); aucane@terra.com.br (A.C.N.); 2 Laboratory of Drug Design (LAPDESF), School of Pharmaceutical Sciences, University of São Paulo State (UNESP), Araraquara 14800-903, SP, Brazil; cauebenitos@gmail.com (C.B.S.); jromanolopes@gmail.com (J.R.L.); jean.santos@unesp.br (J.L.d.S.); 3 Chemical Institute, University of São Paulo State (UNESP), Araraquara 14800-900, SP, Brazil; 4 Faculdade de Medicina de São José do Rio Preto ( FAMERP), Sao Jose do Rio Preto 15090-000, SP, Brazil; 5 Laboratory of Synthesis and Drug Design for Neglected Disease (LAPEN), School of Pharmaceutical Sciences, University of São Paulo (USP), São Paulo 05508-220, SP, Brazil; jeanineg@usp.br (J.G.); elizabeth.igne@gmail.com (E.I.F.)

**Keywords:** Chagas disease, *Trypanosoma cruzi*, resveratrol, curcumin, anti-inflammatory

## Abstract

Chagas disease (CD) is a neglected protozoan infection caused by *Trypanosoma cruzi*, which affects about 7 million people worldwide. There are two available drugs in therapeutics, however, they lack effectiveness for the chronic stage—characterized mainly by cardiac (i.e., cardiomyopathy) and digestive manifestations (i.e., megaesophagus, megacolon). Due to the involvement of the immuno-inflammatory pathways in the disease’s progress, compounds exhibiting antioxidant and anti-inflammatory activity seem to be effective for controlling some clinical manifestations, mainly in the chronic phase. Resveratrol (RVT) and curcumin (CUR) are natural compounds with potent antioxidant and anti-inflammatory properties and their cardioprotective effect have been proposed to have benefits to treat CD. Such effects could decrease or block the progression of the disease’s severity. The purpose of this systematic review is to analyze the effectiveness of RVT and CUR in animal and clinical research for the treatment of CD. The study was performed according to PRISMA guidelines and it was registered on PROSPERO (CDR42021293495). The results did not find any clinical study, and the animal research was analyzed according to the SYRCLES risk of bias tools and ARRIVE 2.0 guidelines. We found 9 eligible reports in this study. We also discuss the potential RVT and CUR derivatives for the treatment of CD as well.

## 1. Introduction

Carlos Chagas was the researcher who first described the Chagas Disease (CD) in 1909. CD, also named American Trypanosomiasis, is an infectious disease caused by the flagellated protozoan *Tryparnosoma cruzi* (*T. cruzi*). Until the year of 2021, no effective and safe treatment focusing on the cure was described, and this neglected disease is now considered a great Health Problem, mainly for some developing countries [1,2,3].

According to the WHO (2022), there are about 6–7 million people infected with *T. cruzi*, worldwide and 75 million are at risk of getting sick [3]. Although most severe cases occur in Latin America, globalization has contributed to the spread of the disease worldwide. Thus, it is not uncommon to find reported cases in developed countries, such as the USA, Canada, and Japan, bringing for those countries concerns that were previously restricted only to poor countries [1,2,3,4]. The transmission of the parasite occurs by the bite of infected bugs (*Hodnius, Triatoma*, and *Panstrogylus*). However, there are other ways of transmission, including blood transfusions, accidents, oral infections, and congenital transmission [4,5,6]. Oral transmission occurs when people eat food or drinks (such as sugar cane juice) freshly prepared and contaminated with vector-infected feces. Such events are associated to high case-fatality rates that can reach up to 33% due to severe acute infection, with a high incidence of myocarditis [6,7,8,9,10]. Most of the infected persons survive with an asymptomatic disease, leading to chronic phase progression over years or decades, which in turn leads to cardiomyopathy and/or digestive manifestations such as megacolon and megaesophagus [3,4,5,6,11,12,13,14].

Cardiac myopathy is characterized by a dilated heart, arrhythmias, an apical aneurysm, thromboembolic events, left ventricular systolic dysfunction, and heart failure, leading to the death [15,16]. Lidani et al. (2020) reported that from 237 patients diagnosed with the infection of *T. cruzi* in the south of Brazil, cardiac (53%) was the predominant form of CD, followed by indeterminate (36%), and digestive (11%), with the average number of comorbidities/patients being 3.9 ± 2.3, including hypertension (64%), dyslipidemia (34%), and diabetes (19%). They also found the influence of the sex of the patient, as the severity of the disease was increased in male patients (2.92 with the highest odds) [17].

Two drugs are available in therapeutics, nifurtimox and benznidazole (BZN), which were discovered in 1965 and 1971, respectively, being active only in the acute phase, without efficacy in blocking the damage progression to the organs [3,4,5,18,19].

Studies show the involvement of the inflammatory and immunological pathways in the progress of the disease, functioning as mechanisms for controlling its manifestation without the total death of the parasites, which persist in the host organism at low levels or in a latent form [20,21,22]. This process promotes progressive pathological damage in organs such as the heart, esophagus, and colon, modifying the architecture and functionality of the affected organs, affecting the quality of life [20,21,22]. 

In biopsies/necropsy, it has been found that the heart is the most affected organ during acute infection [23]. Chagasic chronic cardiomyopathy (CCC) occurs in around 20–30% of patients and the exact mechanism is not well established [24]. Several hypotheses have been reported, including genetic diversity among different strains of *T. cruzi* that might determine different disease evolutions. Additionally, the involvement of immunity may be the basis of the inflammation during the chronic phase of the disease that can lead to CCC [23]. 

The trigger of the inflammatory mechanism, such as innate immunity starting at the acute phase and followed by adaptive immunity through the interleukin IL-12 priming of IFN-producing *T. cruzi*-specific T cells, may be the basis of the inflammation observed in the chronic stage. The production of IL-10 and the transforming growth factor (TGF) inhibits the macrophage trypanocide activity and induces parasite replication and other adaptive immunity mechanisms, including the induction of MYD88/IL12-dependent Th1 cells, a B cell parasite-specific humoral response, CD8+ T-cell persistent responses, and effector mechanisms mediated by interferons (IFN) [25,26].

This inflammation process results in several changes in the microvascular circulation, including perivascular inflammation, changes in the thickness of the capillary basement membrane, an increase in prothrombotic factors, and alterations in vasoconstriction and vasodilatation that can lead to ischemia and tissue necrosis [23].

Due to the capacity in modulating the inflammatory response of the statins, the 3-hydroxy-3-methylglutaryl coenzyme A (HMG CoA) reductase inhibitors, for instance, simvastatin, have been experimentally tested in Chagas disease models [27,28,29,30]. The results were very promising, showing that the drug was able to reduce parasitemia after 26 days post-infection (dpi) and heart damage at the dose of 20 mg/kg/day orally in the Colombian strain in the murine model [27], using treatment for 6 months. Additionally, in infected mongrel dogs, even though the parasitemia was not reduced, a protective effect on the left ventricle ejection fraction, diastolic end diameter, and mass index was shown. Moreover, an increase in the expression of the IL-10 messenger RNA was observed, whereas the proinflammatory cytokine IFN-γ was detected only in infected and untreated animals [30]. The improvement of the cardiac condition lead to a clinical trial of atorvastatin (NCT04984616) [31] and other anti-inflammatory nutrients, such as selenium (NCT00875173) [32] and omega-3 (NCT01863576) [33], demonstrating a new therapeutic focus in the treatment of Chagas disease. 

Resveratrol (RVT) is the polyphenol 3,5,4’-trihydroxy-*trans*-stilbene, a natural compound isolated from the peel of grapes and well known for possessing several beneficial effects to health [34,35], such as anti-inflammatory [36,37] and antioxidant activities [38], action against chronic diseases, including cardiovascular diseases [39,40,41], hypertension [42], kidney diseases [43], diabetes mellitus [44], obesity [45], neurodegenerative diseases (Parkinson’s, Huntington’s, and Alzheimer’s disease) [46,47,48,49,50,51], cancer (breast, colorectal, and multiple myeloma) [52,53,54,55,56,57,58,59,60], and antiviral [61,62,63] and antibacterial activity [64,65]. This variety of effects of RVT is attributed to its interactions and modulations with several biological targets in a complex mechanism, lowering the expression of inflammation markers, including inflammatory cytokines, tumor necrosis factor α (TNF-α), nuclear factor-κB (NF-κB), cyclooxygenase 2 (COX-2), a vascular endothelial growth factor (VEGF), an intercellular adhesion molecule (ICAM), a vascular cell adhesion molecule (VCAM), insulin-like growth factor 1 (IGF-1), insulin-like growth factor-binding protein (IGFBP-3), matrix metalloproteinases (MMPs), 5′-AMP-activated protein kinase (AMPK), caspases, and others, and acting as a multi-target compound [34,35,36,37,38,39,40,41]. In addition, RVT acts by epigenetic pathway, in the SIRT family of deacetylases, which regulates the longevity and increases the life span, thus being considering as a potent anti-aging agent [66,67,68,69].

The cardioprotective effect of RVT may also involve the increase of nitric oxide (NO) production by the increase of NO synthase (eNOS) expression in endothelium cells. This is caused by the overexpression of SIRT-1 [70,71,72,73], which prevents the uncoupling of eNOS, leading to decreasing of superoxide (ROS) production under pathological conditions [73]. In addition, the cardioprotective activity of RVT is associated with the modulation of the composition of gut microbiota which can alter the profile of the host metabolite involved in cardiovascular health [74] and can modulate the biological circadian rhythm [75,76].

Curcumin (CUR) is a natural polyphenol [1,7-bis(4-hydroxy-3-methoxyphenyl)-1,6-heptadiene-3,5-dione] found in the rhizome of *Curcuma longa* (turmeric) and in others *Curcuma* spp. The compound presents several biological activities, including antioxidant and anti-inflammatory, by inhibiting ROS, NF-κβ, TNF-α, and inflammatory cytokines [77,78]. CUR interferes in epigenetic pathways through the inhibition of p300/CREB-specific acetyltransferase, which leads to the repression of the acetylation of histone/nonhistone proteins [79]. CUR, as well as RVT, is considered a pan inhibitor, acting on several therapeutic targets, which can explain its effectiveness in a large range of diseases, mainly in their chronic phase [80,81]. It has been shown to prevent and reverse cardiac hypertrophy and failure in animal models [82,83].

Based on the cardioprotective, antioxidant, and anti-inflammatory effect of RVT and CUR, both of these natural compounds have been tested against *T.cruzi* activity and the protection of cardiomyocytes from damage. As the several attempts to treat CCC with trypanocide drugs have produced inconsistent results, despite reductions in parasite load, the purpose of this work was to search clinical and animal studies of RVT and CUR for the treatment of CD. We set out to answer the following review questions:

⟹ Are RVT and CUR trypanocide agents?
⟹ Are there any RVT and CUR benefits in in vivo-infected animals with *T. cruzi* which can support the clinical study?

In addition, this work aimed to review derivatives of RVT and CUR as potential antichagasic compounds. 

## 2. Methodology

### Literature Search

We have searched for the animal and human studies based on literature published until 30 October 2021 in databanks Pubmed/Medline, Embase, Lilacs, Cochrane Library, and Clinical.trials.gov, using Medical Subject Healing (MESH) terms for Chagas disease, *Trypanosoma cruzi*, resveratrol, curcumin, antioxidant, and anti-inflammatory. The review was performed according to PRISMA guidelines [84] and was registered in PROSPERO (CDR42021293495). In addition, this review includes potential resveratrol and curcumin derivatives proposed for the treatment of CD.

Inclusion criteria: All animal research (male or female, strains of *T. cruzi*, and stage of the disease), clinical trials, case reports, all stages of CD with the intervention of resveratrol and/or curcumin (oral or i.p administration, before or after infection in any time). Only English language was included in the study.

Exclusion criteria: No RVT or CUR intervention, in vitro, ex vivo RVT or CUR intervention, in silico and genetic experiments, comments, editorials, posters, letters, notes, and reviews were excluded. 

Data extraction: Three independent groups of two authors extracted information from the selected articles and, if needed, the differences were resolved by another author. The extracted data included type of studies, and important results and references. The primary outcome was the decrease of parasitemia, and the second was the improvement of heart and digestive function and anti-inflammatory activity. 

Primary outcome: parasitemia (blood or organs parasite load) and/or animal survival.

Second outcome: effects on the organs (heart, brain, liver, esophagus, colon, and others). Inflammation and/or oxidative markers. 

Data and bias analysis: Joanna Brigs Appraisal Critical and Cochrane bias guidelines, and SYRCLE’s risk of bias [85] and ARRIVE 2.0 guideline tools [86,87] for animal studies were used and classified for quality as green (good), yellow (fair), and red (bad). The risk of bias was classified as yes, no, and unclear. If yes, it was classified as low, fair, or high risk.

## 3. Results

### Literature Search and Study Selection

**Figure 1:**
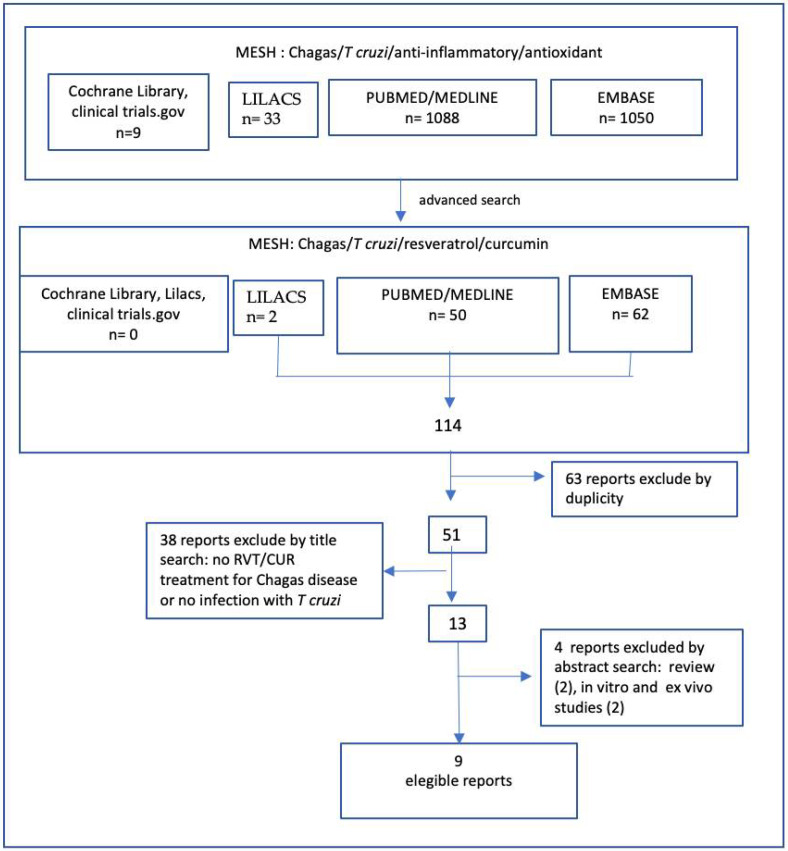
Flowchart of literature search.

The literature search in Pubmed/Medline, Embase, Lilacs, Cochrane Library, and Clinical.trials.gov, using the MESH terms anti-inflammatory, antioxidant, Chagas Disease, and *Trypanosoma cruzi*, found 2180 reports, including 9 clinical trials. The advanced search choosing more specific terms such as resveratrol, curcumin, Chagas disease, and *Trypanosoma cruzi* found 114 reports, of which 63 were excluded due to duplicity and 38 were excluded for having no RVT or CUR intervention or no infection with the parasite, and 4 were excluded because two were reviews and other two showed only in vitro and ex vivo experiments. After exclusion and re-searching in February 2022, 9 records were included in the study, as shown in the flowchart in Figure 1. Table 1 and Table 2 show the results of the selected reports. No clinical study was found and almost all included records were conducted with no randomization or blindness. This is not an experimental practice in animal models. However, it is an important point to evaluate avoiding discrepancies in assessing the methodological quality and bias [85]. In view of this, the works without animal randomization and blindness were considered as half orange in color classification of bias/quality.

## 4. Discussion

Natural products have been used in Medicinal Chemistry as hits and/or leads to be optimized with the aim to discover new agents for many different diseases [97]. This occurs also with neglected tropical diseases, such as Chagas disease [1]. Therefore, biodiversity, that is mainly strictly related to medicinal plants, has been a source of compounds that could turn into drug candidates [98]. Searching for a mechanism of RVT action, in 2012, Vera and colleagues [99] performed docking with arginine kinase, a possible target from *T. cruzi*, using 24 polyphenolic compounds and 18 arginine analogues downloaded from the ZINC database. From those compounds, RVT was chosen for additional tests, considering its ligand efficiency for the binding site of arginine kinase. This compound inhibits 50% of the recombinant arginine kinase activity at the concentration of 325 μM. The IC_50_ observed in *T. cruzi* trypomastigotes-infected CHOK1 cells was 77 μM. Despite its low activity, this compound showed to be promising due to its lack of toxicity and its accessibility, as it is commercially available with a low price. In addition, the selectivity of its target, as it is not found in mammalian hosts, led to its possible use against *T. cruzi*. 

Despite RVT showing trypanocide in vitro activity, based on the results, the RVT failed to decrease *T. cruzi* parasitemia in the acute phase of Chagas disease in vivo. We found four reports involving treatment with RVT in mice infected with *T. cruzi* and only three have parasitemia data [88,89,90,91]. Two of them were the same group of research and showed no significant parasitemia load difference at 8 days post-infection (dpi) with the RVT, 100 mg/kg via gavage, or the control [90,91]. Even though RVT has no trypanocidal activity in vivo in the acute phase, it was shown to decrease ROS in the brain [90] and liver [91], protecting those organs against the inflammatory aggression which was promoted as a defense response from the infection by the parasite. 

Vilar et al. [88] established the chronic parasitemia with the Colombian I strain of *T. cruzi* (60 dpi) and started a 30-day treatment with 15 mg/kg (ip) or 40 mg/kg (per os) of RVT. At 90 dpi, they showed a decrease of around 90% of the heart tissue parasitemia observed, as detected by quantitative PCR, without altering the number of inflammatory cells infiltrating the heart, heart vascularization, or collagen content. They showed that infected mice presented normal ECG profiles after RVT treatment, 15/47 (31%), while the vehicle (VEH) presented cardiac alterations such as sinus arrhythmia, and atrial and/or atrioventricular conduction disorders in 48/48 (100%, *p* < 0.0001). RVT also restored the ejection fraction showing an improvement in the stroke volume and cardiac output compared to the VEH. In addition, RVT activated AMPK phosphorylation and reduced oxidative stress in the heart. These results were not observed in a shorter time of treatment (20 h) or with a lower dose (5 mg/kg). 

In contrast, Wan and co-workers [89] reported that RVT-treated infected animals (90–111 dpi) exhibited a moderate (up to 20%) improvement in the end systolic volume (ESV), stroke volume (SV), and cardiac output (CO) induced by the parasite infection and statistically insignificant improvement in the ejection fraction (EF) and fractional shortening (FS). In addition, RVT exhibited modest control of the left ventricular mass and no improvement in the inter-ventricular septum (IVS), LV posterior wall (LVPW) thickness, or LV area. These results could have occurred because the bias of the treatment chosen by the authors (20 mg/mL in drinking water) did not measure the amount of water drunk by the animal during the time of treatment, making it difficult to control the right concentration given to the animal. In addition, the solubility of RVT in water is very low (0.03 mg/mL) [100]. It was not considered by the authors that the concentration used was probably insoluble and, with the time, the RVT could be precipitated, suggesting an insufficient dose of RVT and a low response. The authors also tested a small SIRT agonist molecule (SIRT1720) which was not RVT-structure related and showed a better response compared to RVT. However, the administration route was different and, because the experiment was not blind, the possibility of bias in this experiment could be increased. CUR, (1,7-bis(4-hydroxy-3-methoxyphenyl)-1,6-heptadiene-3,5-dione (commonly called diferuloylmethane), the other natural bioactive compound, is also a natural phenolic antioxidant, free radical scavenger, acting on the release of superoxide radicals, on nitric oxide in immune cells, and as an inhibitor of lipoperoxidation [101]. Therefore, it is estimated that this phenolic compound acts on modulating signaling molecules, transcription factors, besides some important enzymes, such as protein kinases and protein reductases related to cardiovascular diseases [101]. 

Five reports were included involving CUR treatment and most of them carried out in vivo and in vitro experiments [92,93,94,95,96]. We collected only the in vivo results. The most common problem found was the quality of the CUR from 65% up to 80% purity by High Performance Liquid Chromatography (HPLC). CUR solution preparation was different or not informed by the authors. 

In 2012, Nagajyothi et al. [92] tested the inhibitory effect of CUR on the parasite invasion. The results showed that CUR can decrease the heart parasitemia load compared to the control at 23 dpi and increase the survival rate (100% against 60% in non-treated animals). The authors reported 35 days of CUR treatment (100 mg/kg, orally) and 23 days of infection, suggesting 8 days of pretreatment or a mistake, that was not solved, as we tried to contact the authors with no success. The experiments also showed very good results in decreasing inflammatory markers (heart and liver), suggesting the protection of infection damage. The CUR used was presented as having a > 65% purity and there was no information about the vehicle used.

Novaes et al. [93] performed two different experiments: first, the CUR (100 mg/kg) alone and in association with benznidazol (BZN, 50 and 100 mg/kg), the currently used drug; the second, the same groups with 3 immunosuppression cycles with cyclophosphamide to evaluate infection recrudescence or cure. The results showed that CUR can decrease parasitemia (decrease of parasitemia with BZN100 + CUR > BZN50 + CUR > BZN 100 > BZN 50 >> CUR compared to untreated animals). CUR improved survival, decreasing 25% of mortality (at 24 dpi) against 58.33% of untreated animals after 20 days of treatment. The negative parasitemia animals were selected to perform the recrudescence experiment. Recrudescence after immunosuppression occurred in CUR (75%), BZN50 (66,67%), BZN 100 (25%), but was not observed when CUR was associated to BZN (with both doses, 50 and 100 mg/kg). This methodology is very important to show real antichagasic efficacy on the parasitemia load after treatment, as the immunosuppressor can be reactive, dormant, or in a non/low-replicating (latent) form during the intracellular cycle of *T. cruzi*, improving the quality of the work. The authors also reported a significant reduction (38%) of macrophage infiltration and a large decrease of the heart and liver inflammation markers, such as TNF-α (8000 ↓), at 20 dpi. The purity of the CUR used was not informed and it was suspended in an aqueous solution of 1% carboxymetylcellulose.

The Hernandez’s group conducted the other three researches involving CUR [90,91,92]. In the 2016 [94] and 2018 [95] experiments, CUR presented a purity ≥ 94% for curcuminoids and ≥80% for curcumin (by HPLC), dissolved in corn oil. In the first publication [94], Hernandez et al. treated infected mice (*T. cruzi* RA strain) with different concentrations of CUR (25, 50, and 100 mg/kg) for 35 days. The results showed 100% survival with 100 mg/kg (but not 25 or 50 mg/kg) and 100 mg/kg BZN against 55% of untreated infected mice after 35 dpi. Despite this, the cardiac parasitemia burden was not modified with CUR (all doses) at 21 dpi compared to the non-treated animals observed by quantitative PCR analysis. The second outcome, despite the parasitism, a significant inflammatory process of attenuation was shown in the heart tissue with CUR 100 treatment, analyzed by leukocyte infiltration, cyclooxygenase-2 (COX-2), microsomal prostaglandin E synthase-1 (mPGES-1), and B-type natriuretic peptide (BNP) mRNA expression to a normal (non-infected animal) level. The antichagasic BZN showed almost a 100% decrease on heart parasitism at 21 dpi. However, the inflammation was not attenuated and showed similar results to the infected and non-treated animals. 

In 2018, Hernandez et al. [95] performed the infection of the mice with the *T. cruzi* Tulahuen strain for 14 days. The treatment with CUR (100 mg/kg) or BZN (100 mg/kg) showed that CUR had no effect on decreasing the bloodstream parasite burden, while BZN presented an antiparasitic effect. Even though CUR had no antiparasitic effect, 100% survival was shown after 14 days of infection and there was a significant reduction in the inflammation of the myocardial arteries, decreasing the inflammatory cell infiltration of the heart vessels (histologically analyzed and scored), vascular permeability, and IL-6 and TNF-α mRNA levels in the total heart extracts, compared to BZN and non-treated animals. The histological analysis of the heart was double-blind, conducted of randomized slices. 

Based on the poor solubility of CUR that hinders the bioavailability and the lack of drugs for the chronic phase of CD, Hernandez et al., in 2021 [96], conducted the experiment in the chronic phase with 200 mg/kg of CUR nanoparticules (nano-CUR) for 30 days, starting at 60 dpi, comparing with BZN in a suboptimal dose (25 mg/kg), at 130 dpi. The survival level was not reported and the parasitemia load was detected only in the myocardium, which was shown to be 4.39-fold higher in infected animals and nano-CUR, compared to those treated by BZN, suggesting that nano-CUR has no antiparasitic activity. However, lower CK (creatine kinase) activity was observed, which was not reverted by the current therapeutic drug, BZN. CK is also known as creatine phosphokinase (CPK), a very important protein marker for myocardium damage [102]. The effect of CUR in combination with BZN decreases the level of CK circulating by about 16-fold compared to untreated animals. The authors found an intense reduction of the long-term inflammation in the myocardium, observed by downloading the inflammatory marker levels (IL-1β, TNF-α, IL-6, and CCL5, and heart histopathological analysis with CUR, but not BZN, with the exception of TNF-α). The tissue collagen deposition was decreased in CUR, and was not changed with BZN, but it was more effective when used in combination with the latter drug. These results are very consistent in showing the cardiac benefits of CUR plus BZN [96].

### 4.1. Limitations of the Study

The limitation of the study was the low number of experimental studies in vivo (9 reports), performed with different experimental strains, animals, phases of the disease, period of treatment, and doses, making the comparison difficult. None of works have been executed with randomization/blinding and were considered as half orange. Due to the low solubility of both of the compounds, we found problems with the RVT concentrations and vehicles used for improving CUR that was not uniform. Additionally, the purity of CUR varied with the experiment (found by the informed CAS number), from ≥65% CUR (no total curcuminoids were informed) to ≥94% of curcuminoids and ≥80% of CUR, that can increase the bias of the study. 

### 4.2. RVT and CUR Derivatives

**Figure 2:**
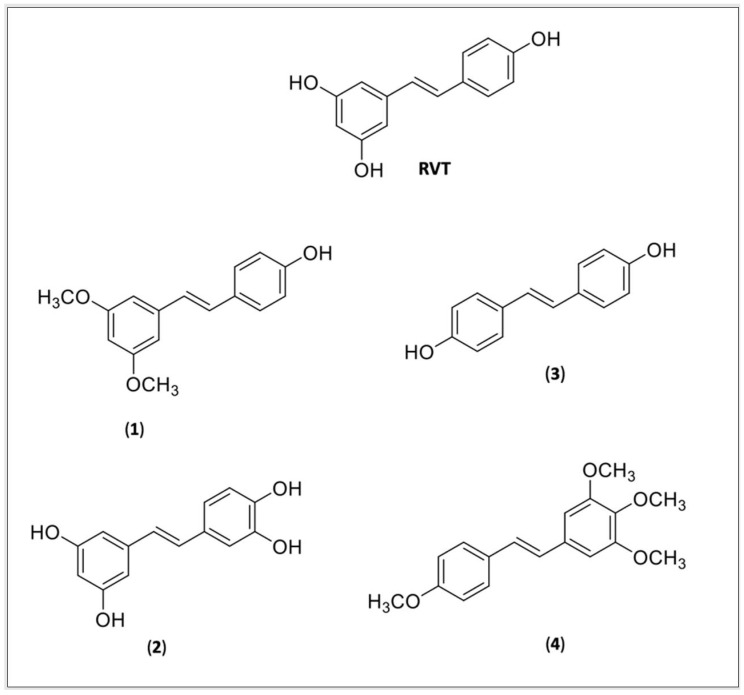
Resveratrol (RVT) and RVT derivatives: (**1**) Pteorstilbene; (**2**) Piceatannol; (**3**) DHS or dihydroxystilbene; (**4**) DMS 212 or TMS, a tetramethoxystilbene.

The absorption, distribution, metabolism, and excretion (ADME) properties are one of the most important barriers that both RVT and CUR found decreased their effectivity. In spite of their lipophilic nature, they showed a low bioavailability due to poor absorption, a fast metabolism, and elimination [103,104]. Several derivatives were synthetized to improve RSV and CUR physicochemical (poor solubility) and pharmacokinetics (poor bioavailability) properties. Most of the compounds were studied for anticancer activity. The methoxylated, hydroxylated, and halogenated RSV derivatives were obtained and showed beneficial biological effects and a potential increased oral bioavailability [99]. None of the compounds were tested for Chaga’s disease, however, some of them were reported to be beneficial for cardiovascular diseases (Figure 2), such as pteorstilbene (**1**), piceatannol (**2**), dihydroxystilbene (**3**), or DHS and a tetramethoxylated derivative (**4)**, DMS 212 or TMS. 

Pterostilbene (**1**) is a natural dimethoxylated analogue of RVT (trans-3,5-dimethoxy-4′-hidroxystilbene) which is reported to have similar activities, including cardioprotective activity [105] and decreasing cardiac oxidative stress [106]. Pterostilbene is 80% bioavailable against 20% of RVT and at concentrations of 1 and 3 uM it prevents myocardiac hypoxia/reperfusion (H/R) or ischemia-reperfusion (IR), and induces H 9c2 apoptosis, against the dose of 20 uM observed by RVT, suggesting it might be more potent than RVT [107]. A docking experiment revealed that pterostilbene interacts with Cys482 and Arg 466 of the active pocket of SIRT-1,2 and its effect is abolished by pretreatment with the SIRT-1 antagonist, the splitomicin [108].

Piceatannol (**2**), is a metabolite of RVT from cytochrome P4501B1 which has similar RVT activity [109], including various cardioprotective effects [110] such as the prevention of cardiac arrythmia, ischemia/reperfusion (I/H) injury in rats, delaying sodium ion current inactivation, showing to be more potent than RVT, strengthening the effective refractor period elongating the action potential in the cardiomyocytes [111]. The study by Wang et al. (2019) showed that (**2**) can protect the heart tissue from peroxidative injury by the upregulation of PI3K-Akt-eNOS (phosphoinositide 3 kinase—protein kinase—endothelial nitric oxide synthase) signaling [112].

Coppa et al. (2011) reported the possible cardioprotective activity mechanism of the DHS (**3**), a dihydroxylate derivative of RVT. They found that (**3**) can inhibit the secretion of endothelin-1 mRNA expression, a vascular tension regulator, and decrease the endothelin-converting enzyme-1mRNA levels, a protein involved in the proteolytic processing of endothelin-1, suggesting that the cardioprotective element is independent of their antioxidant activity [113]. 

A tetrahydroxylmethylated derivative of RVT (**4**) named DMS 212 or TMS (*trans*-3,4,5,4′-tetramethoxystilbene) is a more soluble derivative, presenting faster absorption than RVT because of the presence of one more hydroxy group [105]. Liu et al. [114] showed that TMS can prevent cardiovascular diseases by remodeling H/R induced in pulmonary hypertensive rats through the inhibition of NOX/VPO1 pathway-mediated oxidative stress and the inflammatory reaction.

**Figure 3:**
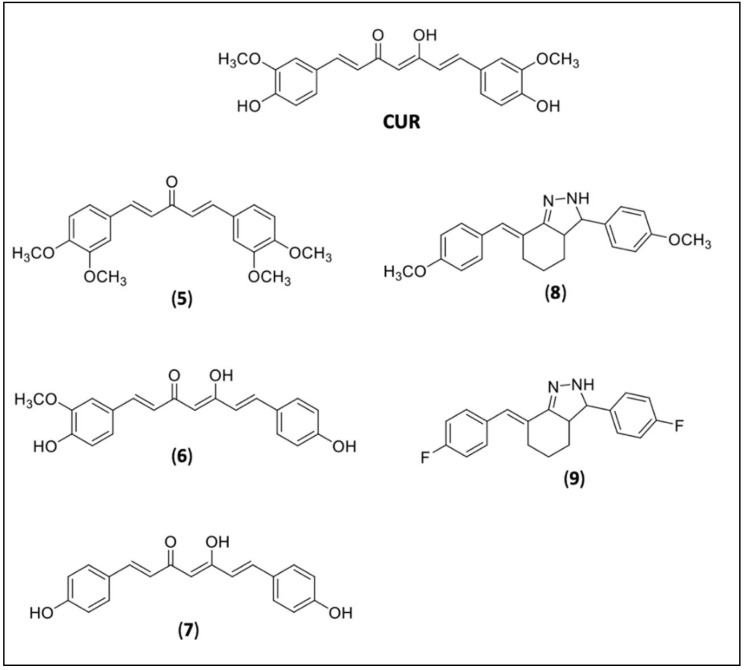
Curcumin (CUR) and CUR derivatives. Natural curcuminoids diveratralacetone (**5**), demethoxycurcumin or DMC (**6**), and bis-demethoxycurcumin or BMC (**7**); synthetic CUR derivative 4a (**8**), 4e (**9**).

The curcuminoids are bioactive components of turmeric (*Curcuma longa*) with a content of around 77% of CUR, 17% of demethoxycurcumin (**6**), 3–6% of bis-demethoxycurcumin (**7**), and others [109]. CUR showed in vitro trypanocidal activity with an IC50 value of 10.13 μM on epimastigotes against 11.07 and 45.33 μM for DMC (**6**) and BMC (**7**) [115]. Due to its low solubility and bioavailability, several efforts have been made to improve it by new formulations or by molecular modifications (prodrug design) [116]. Most of the new derivatives were obtained for *Leishmania*, and only one was found for CD. Matiadis et al. (2021) synthesized fifteen new pyrazol(in)e derivatives of CUR and two of them, the 4a (**8**), (*E*)-7-(4-methoxybenzylidene)-3-(4-methoxyphenyl)-3,3a,4,5,6,7-hexahydro-2*H*-indazole, and 4e (**9**), (*E*)-7-(4-fluorobenzylidene)-3-(4-fluorophenyl)-3,3a,4,5,6,7-hexahydro-2*H*-indazole, showed 16-fold (for 4a) and 6-fold (for 4e) higher potencies against *Tc*TIM (*T. cruzi* triosephosphate isomerase) and epimastigotes of *T. cruzi*. in comparison to their curcuminoid precursors [117] (Figure 3).

## 5. Final Remarks

In summary, the limitation of the study was the low number of experimental research in vivo (9 reports), performed with different experimental strains, animals, phases of the disease, periods of treatment, and doses, which made the conclusion difficult. Most of the works have not been performed with randomization/blinding and were considered as half orange. Due to the low solubility of RVT, we found problems with the concentrations and, with CUR, the problem of different purities, which can increase the bias in the study. The studies showed that RVT did not present antiparasitic activity in the acute or chronic phase of Chagas disease in mice. Despite only four studies being found, they showed beneficial effects for the heart, liver, and brain of the infected mice. The reports focusing on CUR showed antiparasitic activity, however, it was not superior to BZN, the current therapeutic drug. However, when used in combination, CUR enhanced the antiparasitic activity of BZN, a result which was observed in a recrudescence experiment. According to the medicinal chemistry point of view, the design and synthesis of a series of RVT and CUR analogues could bring light into their structure–activity relationship (SAR) as trypanocide, providing the means to optimize the structure of this hit and further lead to obtain a drug candidate with a higher efficiency and better pharmacokinetic properties, maintaining the prototype’s lack of toxicity as well. It is worth noting that most of the derivatives found in the literature were designed for cancer and chronic diseases. We found two new derivatives of RVT with good activity in heart damage in the *T. cruzi* infection and two related to CUR which was more potent against *T. cruzi* in vitro. Despite the low reports in *T. cruzi*-infected animals, we support that both RVT and CUR can be tested as adjuvants in the treatment of CD in a clinical trial with the aim to decrease inflammatory processes of the disease infection’s progression, decreasing heart damage. CUR can also be tested in combination with BZN to block the parasite’s life cycle. We also point to a concern about the correct formulation to guarantee the right concentration without bias.

## Figures and Tables

**Table 1:** Effects of Resveratrol on *T. cruzi*, outcomes, quality, and risk of bias of the included studies.

Reference	Animal Specie	*T. cruzi* Strain	Treatment	Results	Q	B
Vilar-Pereira et al., 2016 [88]	BALB/c Mice female or male (5–7-week age)	1 × 10^2^ blood trypomastigote I Colombian strain	30 days RVT 15 m/.kg/i.p, (vehicle = 10% ethanol/PBS) or 40 mg./Kg (10%ethano/PBS)/daily per os. for 30 days from the establishment of chronic chagasic cardiomyopathy at 60 dpi	At 90 dpi: Primary outcome: RVT decreases heart parasite burden. It was detected by quantitative PCR measured by the relative amount of parasites (TcS18) per host DNA (GADPH) in heart tissue. (Around 90%, *p* < 0.005 compared to vehicle.) Secondary outcome: The cardiac function: RVT had faster heart rate and shorter P wave duration, PR, and QT intervals when compared to vehicle, decrease in 35% of sinus arrhythmia-affected mice (16/45), 49% decrease in the percentage of infected mice affected by atrial and atrioventricular conduction disorders (sinoatrial block, intra-atrial/interatrial block, or second-degree atrioventricular block) (23/47). RVT: peroral (40 mg/kg) obtained the same beneficial results; RVT: short term treatment (20 h) did not present benefits on cardiac function RVT did not significantly alter the number of invading inflammatory cells infiltrating the heart, heart vascularization, or collagen content	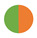	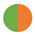
Wan et al., 2016 [89]	C57BL/6 mice Mice (6-weeks-old)	1 × 10^4^ trypomastigotes (SylvioX10 strain, ATCC 50823) were propagated by in vitro passage in C2C12 cells	RVT (20 mg/mL in drinking water) for three weeks, during days 90–111 pi. euthanasia: 150 dpi	Primary outcome: this study did not show parasitemia data. At 150 dpi: Secondary outcome: RVT partially improved the heart function observed by transthoracic echocardiography an overall lackluster performance of RVT in arresting Tc-induced cardiac remodeling and mitochondrial biogenic defects was observed RVT also exhibited modest control of LV mass, but no improvement in the IVS and LVPW thickness and LV area	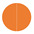	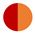
Fracasso et al., 2021 [90]	Swiss mice—female, age not informed	1 × 10^4^ trypomastigote Y strain	RSV (100 mg/kg) compared to BNZ (100 mg/kg) by gavage daily over 7 days.	Primary outcome: RVT has no effect on parasitemia over 8 dpi. Secondary outcome: brains (cerebral cortex) at 8 dpi: RVT decrease ROS levels compared to control (*p* < 0.05). RVT alone or in combination with BZN did not affect lipid peroxidation (TBARS levels) in infected animals (*p* < 0.05) BNZ, RVT, and RVT + BNZ combined downregulated P2X7 expression in the cerebral cortex BNZ and RVT, alone and in combination, up- and downregulated A1 and A2-A receptor densities.	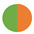	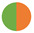
Fracasso et al., 2021 [91]	Swiss mice—female, age not informed	1 × 10^4^ trypomastigotes Y strain	RSV (100 mg/kg (by gavage daily over 7 days.	Primary outcome: at 7 dpi: RVT has no influence on parasitemia (trypomastigote forms) Secondary outcomes: RVT did not revert lipid peroxidation caused by the infection and did not modulate the oxidative stress nor exert effects in antioxidant enzymes RVT decrease SOD activity in infected animals RVT reverses the lower GST in infected animals RVT can downregulate inflammatory response stimulating the expression of NOx	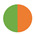	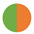

Q: quality were classified as green (good), yellow (fair), and red (bad) according to ARRIVE 2.0 guideline tools [82,83]. B: The risk of bias was classified as yes, no, and unclear. If yes, it was classified as low (green), fair (orange), or high (red) risk according to SYRCLE’s risk of bias [81]. Half orange: no randomization and blinding.

**Table 2:** Effects of Curcumin on *T. cruzi*, outcomes, quality, and risk of bias of the included studies.

	Animal	*T. cruzi* Strain	Treatment	Results	Q	B
Nagajyothi et al., 2012 [92]	Six to 8-week-old male CD-1 mice	Brazil strain (maintained in C3H/He mice)	CUR (100 mg/kg/day orally) for 35 days day of treatment: not informed	Primary outcome: at 23 dpi: decrease of 30% parasitemia compared to infected without treatment heart parasitemia: reduction load in the heart (37% of control, *p* < 0.05) 100% survival against 60% rate in non-treated animals (*p* < 0.05) Secondary outcome: 15 dpi: mRNA levels of NOS-2 (200-fold↑) and superoxide dismutase (Sod1: 7.5-fold ↑) 20 dpi: CUR treatment of infected mice significantly reduced macrophage infiltration to 38% (*p* < 0.05) data compared to curcumin-untreated mice infected with *T. cruzi* (taken as fold 1): Significant reductions in heart and liver inflammation demonstrated a significant reduction in the mRNA levels of inflammatory markers, such as TNF-α (8000 ↓), IL-19 (1300 ↓), IL-22 (3200↓), Bcl2 like1 (6500↓), COX-2 (1038↓), and TLR-9 (4↓) mRNA levels of oxidative-stress-signaling markers in mice hearts: Catalase (912↓) Peroxidase (4000↓), SOD (200↓), NOS-2 (3↓), ApoE (2040↑), NOX-1 (16,854↓), and Myoglobin (20,284↑)	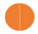	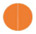
Novaes et al., 2016 [93]	8-week-old Swiss mice (weight, 30.17 ± 3.85 g)	2 × 10^3^ trypomastigotes Y strain	GROUP 1 CUR (100 mg/kg/day orally) CUR + BZN (50 or 100 mg/kg day orally) 20 days of treatment administered after 4 dpi GROUP 2 recrudescence group negative animals of group 1 treated with 50 mg/kg cyclophosphamide in three cycles of four consecutive days with 3 days between cycles	Primary outcome: BZN or BZN + CUR: reduced parasitemia and no mortality compared to untreated group BZN 100 or 50 + CUR showed lower parasitemia compared to BZB only CUR mortality = 25.0%. Untreated: mortality = 58.33% RECRUDESCENCE: Infection recrudescence occurred in 75.0% of the animals in the CUR group. In the BZN50 and BZN 100 groups, the recrudescence rates were 66.67% and 25.0%, respectively. Recrudescence was not identified in the BZN 100 or 50 + CUR groups	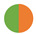	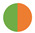
Hernandez et al., 2016 [94]	Six- to eight-week-old female BALB/c mice	50 trypomastigotes of RA strain	CUR, 25, 50, or 100 mg/kg body weight/day orally or BZN, 100 mg/kg body weight/day orally for 35 days.	Primary outcome: 21 dpi: cardiac parasitemia: it was not modified with CUR (all doses) against almost 100% decrease of heart parasitism load 35 dpi: 100% survival with 100 mg/kg (but not 25 or 50 mg/kg) and BZN 100 mg/kg against 55% of untreated infected mice Secondary outcome: 21 dpi inflammatory process attenuation in heart tissue with CUR 100 treatment, analyzed by leukocyte infiltration, cyclooxygenase-2 (COX-2), microsomal prostaglandin E synthase-1 (mPGES-1), and B-type natriuretic peptide (BNP) mRNA expression to normal (non-infected animal) level	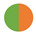	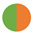
Hernandez et al., 2018 [95]	C5BL/6 male mice (eight weeks old)	103 blood trypomastigotes of Tulahuen strain	CUR (100 mg/kg) dissolved in corn oil oral (by gavage) from day 1–14 of infection BZN 100 mg/kg same period	Primary outcome: parasitemia 14 dpi: CUR very little effect on parasitemia profile compared to non-treated animals Secondary outcome: significant reduction of inflammation of myocardial arteries: observed by the significant decrease of the inflammatory cell infiltration of heart vessels (histologically analyzed and scored), vascular permeability, and IL-6 and TNF-α mRNA levels in total heart extracts by CUR.	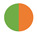	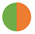
Hernandez et al., 2021 [96]	C57BL/6 mice female and male mice (eight weeks old)	10,000 Brazil strain (DTU I, routinely maintained by serial subinoculation in C3HeJ mice at three-week intervals)	Nano formulated Cur preparations (size range, 250–300 nm) contained 0.15 mg CUR per mg of polymer, suspended in an aqueous solution of 1% wt/vol sodium carboxymethylcellulose and administered orally by gavage (0.15 mL) once a day. Treatment for 30 consecutive days, starting on day 60 of infection.	Primary outcome: at 130 dpi survival: heart tissue parasitemia: T. cruzi load was 4.39-fold (*p* < 0.01) higher in the myocardium from mice administered with PBS than in those treated with BZ Cur therapy had no significant effect on cardiac parasitism and did not limit BZ parasiticidal activity. Secondary outcome: at 130 dpi CK activity: Chronic Chagas mice receiving BZ or CUR showed lower (*p* < 0.01) CK activity than that recorded in untreated animals. BZN + CUR + 16-fold decrease in circulating CK values (*p* < 0.001 vs. untreated infected mice). CUR reduce circulating ANP levels alone or in combination with BZN. BZN alone does not revert ANP serum elevation CUR reduce intensity of long-term inflammation in myocardium (IL- 1β, TNF-α, IL-6, and CCL5) but not by BZN (exception for TNF-α) BZN is unable to impair leukocyte influx CUR + BZN enhances cardioprotective effect	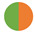	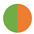

Q: quality were classified as green (good), yellow (fair), and red (bad) according to ARRIVE 2.0 guideline tools [82,83]. B: The risk of bias was classified as yes, no, and unclear. If yes, it was classified as low (green), fair (orange), or high (red) risk according to SYRCLE’s risk of bias [81]. Half orange: no randomization and blinding.

## Data Availability

Not applicable.
